# *mebipred*: identifying metal-binding potential in protein sequence

**DOI:** 10.1093/bioinformatics/btac358

**Published:** 2022-05-27

**Authors:** A A Aptekmann, J Buongiorno, D Giovannelli, M Glamoclija, D U Ferreiro, Y Bromberg

**Affiliations:** Department of Biochemistry and Microbiology, Rutgers University, New Brunswick, NJ 08873, USA; Institute of Marine and Coastal Sciences, Rutgers University, New Brunswick, NJ 08901, USA; Division of Natural Sciences, Maryville College, Maryville, TN 37804, USA; Institute of Marine and Coastal Sciences, Rutgers University, New Brunswick, NJ 08901, USA; Department of Biology, University of Naples Federico II, Naples, Italy; Institute for Marine Biological Resources and Biotechnology—IRBIM, National Research Council of Italy, CNR, Ancona, Italy; Department of Earth and Environmental Sciences, Rutgers University, Newark, NJ 07102, USA; Protein Physiology Lab, Departamento de Quimica Biologica, Facultad de Ciencias Exactas y Naturales, Universidad de Buenos Aires-CONICET-IQUIBICEN, 1428 Buenos Aires, Argentina; Department of Biochemistry and Microbiology, Rutgers University, New Brunswick, NJ 08873, USA

## Abstract

**Motivation:**

metal-binding proteins have a central role in maintaining life processes. Nearly one-third of known protein structures contain metal ions that are used for a variety of needs, such as catalysis, DNA/RNA binding, protein structure stability, etc. Identifying metal-binding proteins is thus crucial for understanding the mechanisms of cellular activity. However, experimental annotation of protein metal-binding potential is severely lacking, while computational techniques are often imprecise and of limited applicability.

**Results:**

we developed a novel machine learning-based method, *mebipred*, for identifying metal-binding proteins from sequence-derived features. This method is over 80% accurate in recognizing proteins that bind metal ion-containing ligands; the specific identity of 11 ubiquitously present metal ions can also be annotated. *mebipred* is reference-free, i.e. no sequence alignments are involved, and is thus faster than alignment-based methods; it is also more accurate than other sequence-based prediction methods. Additionally, *mebipred* can identify protein metal-binding capabilities from short sequence stretches, e.g. translated sequencing reads, and, thus, may be useful for the annotation of metal requirements of metagenomic samples. We performed an analysis of available microbiome data and found that ocean, hot spring sediments and soil microbiomes use a more diverse set of metals than human host-related ones. For human microbiomes, physiological conditions explain the observed metal preferences. Similarly, subtle changes in ocean sample ion concentration affect the abundance of relevant metal-binding proteins. These results highlight *mebipred’*s utility in analyzing microbiome metal requirements.

**Availability and implementation:**

*mebipred* is available as a web server at services.bromberglab.org/mebipred and as a standalone package at https://pypi.org/project/mymetal/.

**Supplementary information:**

Supplementary data are available at *Bioinformatics* online.

## 1 Introduction

Proteins bind a diverse set of metal ion-containing cofactors to sustain the functional requirements of life. Metal ions, e.g. iron, magnesium, copper, etc., and metal-containing ligands, e.g. heme and iron–sulfur clusters, participate in protein folding/stability (Arnold and Zhang, 1994), DNA replication (Batra *et al.*, 2006), catalysis (Bennett, 1973), redox chemistry (Bennett, 1973) and many other cellular activities. Proteins could thus be described as sophisticated electron transfer nanomachines that depend on transition metal ions to perform their functions (Falkowski, 2015). Of the proteins whose 3D structure is available in the Protein DataBank (PDB) (Bernstein *et al.*, 1977), roughly a third (49 996 of 152 346) are metal-binding proteins, an observation which may, but does not necessarily, reflect their high abundance in nature. Overall, it appears that only a small fraction of metal-binding protein sequences have been identified. The Swiss-Prot (2021) database, for example, contains over half a million manually curated protein sequences, of which ∼14% (94 720) are annotated as metal-binding; specific of the binding activity of only a few of these (<1%, 4251 proteins) has thus far been experimentally verified (Feb 2020). Furthermore, of the nearly 180 million proteins in TrEMBL, generated via translation of sequenced genome open reading frames (ORFs) and having no experimental annotations, only about 5 million sequences, i.e. <3%, are predicted to be metal-binding (UniProt Consortium, 2019).

Different levels of protein redundancy in distinct databases may be an underlying cause for this difference in fractions of metal-binding proteins. However, another major reason is that we are still unable to accurately identify metal-binding proteins directly from their sequences and, in some cases, even from their high-resolution structures (Whittaker, 2003). Experiments, e.g. mass spectrometry (Deng *et al.*, 2010) and crystallography (Handing *et al.*, 2018), can detect protein–metal interactions, but these analyses are expensive and time-consuming, as well as error-prone for both technical and biological reasons. For example, cambialistic proteins can use metal cofactors interchangeably (Lancaster *et al.*, 2004) and thus are likely to be misclassified when experimentally assessed for binding of specific metals. Similarly, some experiments use non-native metals for technical and/or crystallization purposes (Laganowsky *et al.*, 2011), lose record of metal ion-binding ability/specificity in the process of protein purification (Goto *et al.*, 2000) or even simply incorrectly identify the bound metals due to low experimental resolution (Chaudhuri *et al.*, 1999). Thus, only a small portion of extant metal-binding protein sequences have likely been identified.

There is no simple way to establish from sequence whether a protein binds a metal or not, but there have been multiple attempts to predict binding of single ion ligands (including metals) from protein 3D structure. While a complete account of all relevant methods present in the literature is beyond the scope of this work [for a review see Lavecchia and Di Giovanni (2013)], here we highlight some trends in method development.

metal-binding sites in proteins frequently comprise a shell of hydrophilic residues that can be identified in protein structure (Yamashita *et al.*, 1990). For example, one algorithm (Nayal and Di Cera, 1994) detects Ca^2+^ binding via identification of Ca^2+^ ion coordination by a layer of oxygen atoms supported by an outer shell of carbon atoms. Available structure-based methods thus use the knowledge of hydrophilic shell residues to make predictions (Babor *et al.*, 2008; Lin *et al.*, 2016; Nayal and Di Cera, 1994; Un *et al.*, 2004; Yamashita *et al.*, 1990). The main disadvantage of these approaches is that many such hydrophilic shells do not bind metals (Gregory *et al.*, 1993). Additionally, structure-based methods are limited by the relatively small number of experimentally determined protein structures available for analysis (Bernstein *et al.*, 1977). When a protein structure is available, however, these methods often attain better performance than ones based on sequence alone.

To circumvent the limitation in the number of 3D structures, methods using homology modeling of proteins were developed. Early attempts at this type of prediction, e.g. MetSite (Sodhi *et al.*, 2004), had poor performance (58% precision at 28% recall). Overall, methods based on homology modeling tend to perform poorly when predicting sequences modeled with structural templates of <40% sequence identity; e.g. 42% precision at 65% recall as per Levy *et al.* (2009). Moreover, these methods attain a better performance when focusing on a single metal ion than when trying to describe binding of multiple ions, e.g. Liu *et al.* calcium-binding site predictor (99% precision at 75% recall) (Liu and Altman, 2009) and Zhao *et al.* zinc-binding predictor (90% precision at 72% recall) (Zhao *et al.*, 2011).

The computational prediction of metal-binding can be similar in essence to the prediction of other functional characteristics of proteins from sequence, e.g. mutation effects (Bromberg and Rost, 2007), residue importance (Miller *et al.*, 2019) or subcellular localization (Goldberg *et al.*, 2014). Here, evolutionary profiles, predicted structure, physicochemical properties and sequence descriptors are combined as features for machine learning. One such approach to the prediction of metal-binding (Lu *et al.*, 2006) has attained fairly high accuracy (70% overall accuracy). Other methods combine structural and sequence features, e.g. Lin *et al.* (2016) report accuracies above 92%. Combining sequence, structure and residue contact features in a random forest framework, the tool MetalExplorer (Song *et al.*, 2017) predicts the binding of eight metal ions. Performance across ions is varied, with a precision of 60% for recalls ranging from 59% to 88%.

There are also structure-independent (purely sequence-based) methods to predict metal-binding. Function transfer by homology, i.e. the assumption that similar sequences perform similar functions, is one of the simplest ways to infer metal-binding for protein sequences. Similarity is easily established by alignment methods. However, a well-defined alignment score cutoff for identifying functionally similar proteins has yet to be established (Mahlich *et al.*, 2018). Moreover, sequence similarity, or even well-characterized homology, may be misleading as homologs can evolve to bind different metals due to changing environmental pressures (Capdevila *et al.*, 2017). It is also possible to predict metal-binding using sequence conservation of residues near those directly interacting with Zn^2+^, Cu^2+^, Fe^2+^, Fe^3+^ and Co^2+^ ions with a high accuracy (Cao *et al.*, 2017); proteins binding other ions were not identified using this method. Pattern recognition [e.g. hidden Markov models, HMMs (Bateman *et al.*, 2002) and regular expressions, e.g. Andreini *et al.* (2004)] can also be used to expand the suspected set of metal-binding sequences on the basis of remote homology. Unfortunately HMMs, designed to identify evolutionary conserved sequence patterns, are too specific and, thus, not well-suited for *de novo* metal-binding prediction.

More complicated sequence-based metal-binding predictors often use machine learning techniques [e.g. neural networks (Nakata, 1995), support vector machines (Passerini *et al.*, 2006, 2007) and random forests (Kumar, 2017)]. The performance of these methods varies; e.g. Lin *et al.* (2005) reported high precision for all ions, albeit at recall as low as 35%. Combining different methods to identify specific residues involved in metal-binding, e.g. Zn-binding cysteines and histidines, also produced high accuracy (Passerini *et al.*, 2006). Note that while all the above methods report good performance, we were unable to validate these reports using our own data as the webserver/standalone versions (where applicable) were non-functional and downloadable scripts were absent.

Here we present *mebipred* (metal-binding predictor), a computational method for the prediction of protein metal-binding potential based on sequence information alone. Our method is widely applicable because it does not depend on the existence of a high-resolution structure, has a better performance (area under the precision/recall curve = 0.91) and is faster (17 000 sequences/minute) than existing alignment-based tools, and can be used to predict metal-binding using whole protein sequences as well as short peptide fragments. The latter ability makes it potentially suitable for annotation of shotgun-sequenced unassembled metagenomic data/reads. *mebipred* is also alignment-free and, thus, useful for the analysis of newly identified proteins with no known homologs. Finally, as mentioned previously, *mebipred* is the only currently publicly available method for sequence-based prediction of metal-binding.

## 2 Materials and methods

### 2.1 Datasets

We explored proteins binding Na, K, Ca, Mg, Mn, Fe, Cu, Ni and Zn metal-containing ligands, regardless of their oxidation state (e.g. Fe^2+^ and Fe^3+^ are both in the Fe class) or context (e.g. Fe-containing hemes are in the same class as Fe ions). We retrieved all protein structures with these metal-containing ligands from the PDB (July 2019) and parsed them using the BioPython PDB module (Hamelryck and Manderick, 2003). One naive approach to identify a set of metal-binding proteins is to compile all structures that have a metal ion. However, in the case of heteromers, i.e. protein complexes that contain multiple non-identical chains, it is possible that only one of the chains binds the metal. We thus considered as metal-binding only the amino acid sequences/chains with at least one heavy atom within 5 Å of the metal ion (METAL set). All other chains were included in the NO_METAL set, along with all PDB structures that contained no metals at all. Note that this criterion for the differentiation of metal-binding/non-binding chains could lead to disagreement with existing metal-binding annotations (Supplementary Data: PDB_chain_MB_5.0A).

For the METAL set of proteins, we further identified the specific metal ion that the protein bound. These were added as positives to the specific ion (Na, K, Ca, Mg, Mn, Fe, Cu, Ni or Zn) -set; all other proteins, metal-binding or not, were added to the negatives set for that ion (Supplementary Data: PDB_chain_<METAL>_5.0A).

We clustered all (metal-binding and non-binding) sequences at 70% identity using CD-HIT (Fu *et al.*, 2012) (Supplementary Table S1). We decided to use 70% sequence identity as a threshold for clustering because sequence functionality quickly diverges below this threshold (Devos and Valencia, 2000), while protein families can be defined at around this sequence identity as well (Todd *et al.*, 2001). Note that earlier studies have considered lower cutoffs for defining similarity of metal-binding proteins, e.g. 30% and 50%, respectively, for Cao *et al.* (2017) and Kumar (2017). However, whether a specific level of sequence identity constitutes a good proxy for homology (Pearson, 2013) is debatable and beyond the scope of this study. We further considered clusters containing more than 98% of sequences from the METAL set to be positive (5333 clusters), and those containing 98% of sequences from the NO_METAL set to be negative (28 578 clusters). Thus, most of the clusters (∼81%; 33 911 of 42 085) were either in the METAL or NO_METAL set (Supplementary Fig. S1). We retained the MIXED sequence clusters not used in training for testing purposes. We similarly defined the specific ion-binding clusters using the 98% content cutoff, e.g. K-binding positives if 98% of the sequences in the cluster are K-binding and not K-binding negatives if 98% of the sequences in the cluster are not K-binding.

### 2.2 Feature extraction

To describe the proteins in our METAL and NO_METAL sets, we used only sequence-based features: (i) amino acid composition, (ii) amino acid physicochemical properties and (iii) a count of the metal-binding amino acid 5mers (220 features total; Supplementary Figs. S2 and S3).

### 2.3 Machine Learning

Using the above features, we trained a feed-forward multi-layer perceptron with back-propagation using the Keras (Chollet, 2017) implementation in the machine learning framework Tensorflow (Abadi *et al.*, 2016). Our model is a sequential network with the RMSprop (Dauphin *et al.*, 2015) optimizer and a learning rate (lr) = 0.000005. For the optimization of the learning rate parameter, we started with an lr = 0.5, reduced it by an order of magnitude in each iteration of training and set the value to the one that minimizes the loss (calculated as binary cross-entropy). We only optimized the learning rate (on the training set). All other parameters were set at default values according to the Keras manual (Chollet, 2017). Each model was trained for 1000 epochs (stopping time selected based on previous experience with similar datasets). The input layer consisted of 219 nodes—one node per feature. There were two hidden layers, as these are sufficient to approximate most partition problems and require less computational power than more hidden layers (Huang, 2003). Each layer had 219 nodes with a rectified linear unit activation function (or “ReLU”) and a dropout of 0.2. Finally, there was a single-node output layer, using the sigmoid activation function and a default prediction (yes/no) cutoff set at 0.5.

We trained and tested our model for identifying metal-binding proteins using 10-fold cross-validation as follows: (i) we split our set of METAL (positive) and NO_METAL (negative) sequence clusters to create 10 equally sized groups, with 50% positive and negative sequences, each; (ii) we then built 10 models by rotating through the 10 splits, using one group for testing while training with the other nine groups. This cross-validation was used to estimate the performance of the method; the final *mebipred* model was constructed using all positive sequences and an equal number of negatives.

We followed the same protocol for each metal ion model using the respective positive and negative data (Supplementary Data: Positives). For these, we added one more feature to out input set—the score of the general metal-binding model above.

### 2.4 Performance metrics

To measure the performance of our method we calculated overall accuracy, as well as positive precision, recall and F-measure (Equation 1). True positives (TP) are metal-binding proteins predicted as metal-binding, false positives (FP) are metal non-binding proteins predicted as metal-binding, false negatives (FN) are metal-binding proteins predicted as metal non-binding, and true negatives (TN) are metal non-binding proteins predicted non-binding.

(1) $$\begin{array}{c} \mathrm{Precision}=\frac{\mathrm{TP}}{\mathrm{TP}+\mathrm{FP}}\;\mathrm{Recall}=\frac{\mathrm{TP}}{\mathrm{TP}+\mathrm{FN}}\\ \mathrm{Accuracy}=\frac{\mathrm{TP}+\mathrm{TN}}{\mathrm{All}\;\mathrm{predictions}}\\ F1=2\times \frac{\mathrm{Precision}\times \;\mathrm{Recall}}{\mathrm{Precision}+\mathrm{Recall}} \end{array}.$$

*Comparing model performance to existing tools.* To compare our method to a simple alignment-based approach, we extracted all sequences from the PDB. We generated a database of these sequences using the makeblastdb (-blastdb_version = 5 and no extra parameters). We then ran BLAST (ncbi-blast+ V. 2.10.4) (Altschul *et al.*, 1990; Camacho *et al.*, 2009) with default parameters (eval 1; max_target_seqs 1000000) for all-to-all comparisons of protein sequences in this database. We used as gold standard our METAL set, i.e. any sequence that aligned to a protein from the METAL set with a score better than threshold (range e-val = [10^−20^,1] in steps of two orders of magnitude) was considered to be metal-binding. For each e-value threshold, we counted the number of TPs (metal-binding proteins aligning to other metal-binding proteins), FPs (metal non-binding proteins aligning to metal-binding proteins) and FN (metal-binding proteins not aligning to any other metal-binding proteins). Note that since we wanted to evaluate the use case where an unknown sequence is being annotated, we excluded self-hits from BLAST results but did not exclude hits to homologous sequences.

We further compared our performance to that of multiple published tools. For MetalDetector2 (Table 2), we used a set of non-redundant metal-binding PDB structures described as the evaluation set of that method’s manuscript (Passerini *et al.*, 2011) (extracted in Dec 2011; 2982 proteins, 1340 metal-binding). We also compared *mebipred* to two sequence-based methods (Cao *et al.*, 2017; Kumar, 2017) and a structure-based method (MIB) (Lin *et al.*, 2016) using the data from the BioLip database (Yang *et al.*, 2013) (105 152 proteins, 23 094 metal-binding, non-redundant at 90% sequence identity).

**Table 2.** *mebipred* performance versus MetalDetector2

		Precision(%)		Recall(%)
Ligand	*N*	MetalDetector2	*mebipred*	
Zn	817	63	90	70
Fe(Heme)	234	67	93	77
Fe(Fe-S)	202	68	97	67
Cu	87	57	96	64

*Note:*We report Heme and Fe-S performance separately although both methods predict Fe binding without further specification.

### 2.5 Generating short peptides

We fragmented all ≥50-residue protein sequences in the PDB (445 763 sequences) into 50-residue fragments, using a sliding window of one (101 054 024 fragments total).

### 2.6 Metagenomic sample processing

To analyze metagenomic samples, reads were trimmed with trimmomatic (Bolger *et al.*, 2014) using default parameters. Trimmed reads were filtered with phred (Ewing and Green, 1998) using a score cutoff of 28. Reads were then analyzed in two ways:

1. All reads were translated into peptides within the six reading frames using Biopython’s (Cock *et al.*, 2009) standard bacterial codon table. Translated reads of ≤15 amino acids were discarded. Remaining reads were used as input to *mebipred.*
2. Reads were assembled using metaSPAdes (Nurk *et al.*, 2017) with variable kmer sizes (*k* = 21, 33, 55, 77, 99 or 127). ORF calling and translation for the resulting contigs was done by Prokka (Seemann, 2014). Resulting peptide sequences were used as input to *mebipred*.

## 3 Results and discussion

### 3.1 Available metal-binding protein structures are not very diverse

The high-resolution structure of most proteins is not yet available, although this may change soon (Jumper *et al.*, 2022). If a protein is of particular interest for the scientific community, it might be overrepresented in the PDB; e.g. >1300 structures of the SARS-COV2 spike protein. Thus, whether the known protein structures are representative of all naturally occurring proteins is debatable and outside the scope of this work (Jaroszewski *et al.*, 2009). However, available structures constitute the most reliable set of metal-binding proteins (Andreini *et al.*, 2013; Putignano *et al.*, 2018). A third (49 996 of 152 346) of the PDB entries contain at least one of the metal atoms considered here; corresponding to 106 508 metal-binding sequences of 445 763 total. Removing 100% identical sequences further reduces this number to 30 217 metal-binding sequences of 102 479 total (Supplementary Data: Positives).

These 102K sequences can further be clustered at 70% sequence identity (Fu *et al.*, 2012) into 40 850 clusters (representing 39 066 structures) of which only 9% (3542 structures) have at least one sequence from the METAL set. Note that a single structure can bind multiple different ligands and is likely to contain more than one chain binding a certain ion.

### 3.2 *mebipred* attains exemplary performance

**Fig. 1.**
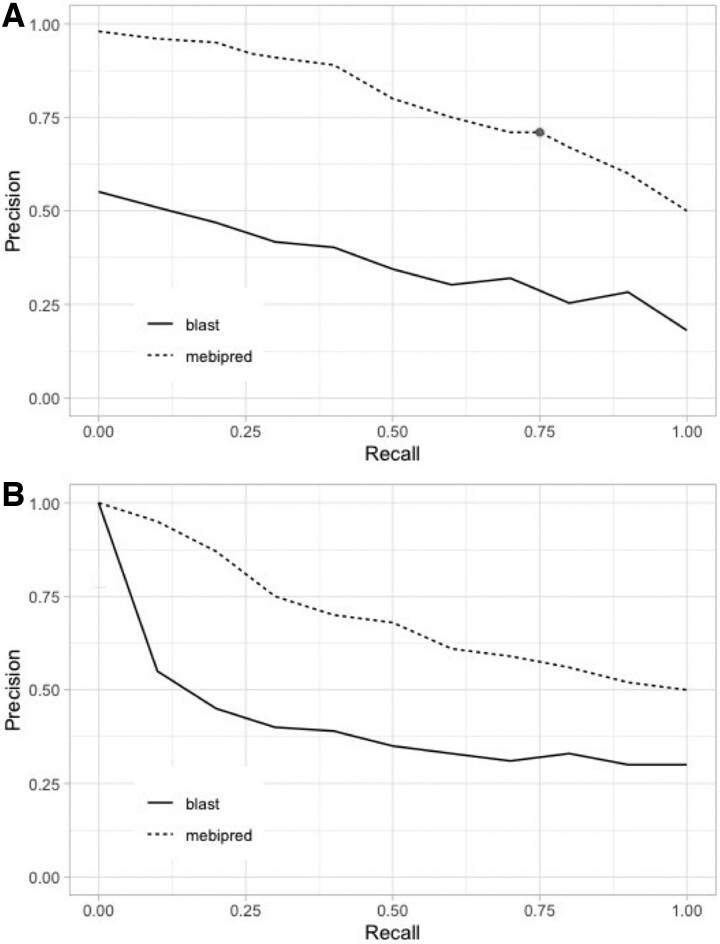
*mebipred* outperforms BLAST in identifying metal-binding proteins and peptides. (**A**) At all cutoffs, *mebipred* (MBP; dashed line) is more precise than BLAST (solid line). For example, at the default cutoff (score = 0.4; black dot) it achieves 71% precision at 75% recall, as compared to 29% precision attained by BLAST at a similar recall. (**B**) *mebipred* also outperforms BLAST in identifying the metal-binding propensity of proteins from their 50 amino acid fragments. For example, for half of the fragments, it attains 67% accuracy, as compared to 35% attained by BLAST

In cross-validation, the first tier model of *mebipred* identified non-redundant metal-binding proteins (binary yes/no) with nearly 92% precision at 26% recall (at 0.5 cutoff)—more than twice the precision obtained by BLAST at a similar recall on the same dataset (Fig. 1A). We then calculated F1_max_ = 0.73 (Materials and methods; precision = 0.71 and recall = 0.75; Equation 1; Table 1), defining a new default cutoff = 0.4. Note that we also evaluated using a RandomForest classifier instead of a Neural Net, but its performance (AUPRC of 0.42 versus 0.83) was worse than that achieved by *mebipred* (Supplementary Fig. S4).

**Table 1.** *mebipred* performance across metals

ANN	AUROC	AUPRC	Prec^a^	Rec^a^	F1^a^
metal-binding	0.91	0.83	0.71	0.75	0.73
Fe	0.95	0.95	0.96	0.91	0.94
Ca	0.86	0.91	0.91	0.77	0.83
Na	0.83	0.83	0.86	0.68	0.76
K	0.91	0.91	0.88	0.84	0.86
Mg	0.82	0.82	0.79	0.8	0.8
Mn	0.91	0.91	0.89	0.83	0.86
Cu	0.97	0.97	0.98	0.92	0.95
K	0.91	0.91	0.88	0.84	0.86
Co	0.85	0.85	0.89	0.71	0.79
Ni	0.91	0.86	0.84	0.67	0.75
Zn	0.9	0.92	0.95	0.7	0.8

a AUCs, F1, Precision, and Recall are reported at a cutoff = 0.4 for the first (metal-binding, MB) tier and at cutoff = 0.5 for the second tier of per ion *mebipred* predictions; in both cases, the default cutoffs are established via F1_max_. At the cutoff = 0.5, the first tier model attains 0.92 precision at 0.26 recall (see Supplementary Data: AUPRCTraining_folds for per-fold performance).

Furthermore, performing the BLAST search for all sequences in the PDB took ∼6 weeks (445 763 chains in 152 346 structures), ∼7.25 s/sequence on average on one core of a 2.4-GHz machine with 16G RAM. The same dataset was processed by *mebipred* on the same machine in 29 min (∼3.5 × 10^−3 ^s/seq). While both BLAST and *mebipred* can run on multiple cores, the difference in speed is likely to be retained. That is, BLAST compute time is expected to grow both with database size and the number of queries (Kent, 2002), while *mebipred* prediction time only reflects the number of queries, i.e. the algorithm scales as *(O)n*.

Finally, we evaluated *mebipred* on the NO_METAL (34 610 randomly selected from 110 140 sequences) and METAL (34 610 sequences) proteins from MIXED clusters excluded from training (balanced set; Supplementary Fig. S5, identifiers and sequences in Additional Data: positive/negative.txt and pdb_seqres.fasta). For this set of previously unseen proteins, our model attained an F1_max_ = 0.74, AUPRC = 0.72 (as compared to F1_max_ = 0.73, AUPRC = 0.83 for the cross-validation evaluation); F1_max_ for this set was attained at the *mebipred* cutoff = 0.4, i.e. as previously selected. Note that the metal-binding proteins in this set are harder to identify because of their inherent (i.e. MIXED cluster) similarity to non-metal-binding sequences. We also compared performance across sequences in this set at different degrees of identity to the training set (Supplementary Table S2). We found that *mebipred* predictions generalize well, with performance across the different sequence-identity datasets varying by no more than a few AUPRC percentage points.

The second tier of *mebipred* models predicts protein binding to a ligand that contains specifically one of the 11 ions under consideration. In cross-validation (Materials and methods), *mebipred* was accurate in predicting ion specificity of individual proteins (Table 1). Note that we did not build predictors for proteins binding other biologically active metals (e.g. vanadium, molybdenum, titanium, etc.), because the number of available structures binding these was insufficient to train a model of this kind. These could be incorporated into *mebipred* in the future if more metal-binding protein structures are resolved.

Note that the predictions of the second tier of *mebipred* do not always match those of the first tier. A metal-binding prediction can still be true in the absence of the specific ion prediction; i.e. a protein can bind metals that are not part of our ion collection. A different type of discrepancy is when the protein is predicted to not be metal-binding, while the second predictor tier identifies a specific ion preference. We evaluated the second tier’s ability to predict metal-binding by considering any positive ion binding prediction (at the default cutoff = 0.5) as an indication of metal-binding. This approach has a precision of 0.38 and a recall of 0.8; increasing stringency to cutoff of 0.9, improves performance (precision = 0.8, recall = 0.78). For comparison, the first tier at the default cutoff of 0.4 has the same precision and a lower recall of 0.5 (Table 1). These observations suggest that in cases of disagreement between the tiers, high-scoring predictions of the second tier can be trusted to identify metal-binding proteins.

Our evaluation of *mebipred* performance against that of other methods on our data was complicated by the absence of working web servers/standalone packages. Thus, we ran our tool on the data used for testing by the different methods. *mebipred* predicted metal–ligand binding better than MetalDetector2 (Passerini *et al.*, 2011) (Table 2), a tool predicting metal-binding sites. It also outperformed methods described in Cao *et al.* (2017) and Kumar (2017) (Table 3), but did worse than structure-based MIB (Lin *et al.*, 2016). We were unable to compare *mebipred* performance to that of MetalExplorer (Song *et al.*, 2017) due to unavailability of either the method or its benchmark dataset. Note that here we used the measure of accuracy to describe performance (Equation 1) since it was reported in the corresponding publications, but precision and recall might be more relevant for imbalanced datasets (Ferri *et al.*, 2009). We also note that the sequence overlap between *mebipred’*s training data and testing sets of other methods may limit this performance evaluation. However, as the definition of metal-binding proteins differs between methods, e.g. we only consider chains in direct contact with a metal to be binding instead of all chains in the structure, we do not expect that *mebipred’*s performance is consistently overestimated.

**Table 3.** *mebipred* accuracy versus other methods

Ligand	Cao *et al.* (2017)	Kumar (2017)	MIB (Lin *et al.*, 2016)	*mebipred*
	Sequence	Sequence	Structure	Sequence
Ca	74.8	75.4	94.1	86.7
Co	83	85.3	94.7	86.2
Cu	96.3	78.1	95.3	87.2
Fe2	91.3	75.6	95.1	89.2
Fe3	87.8	74	94.9	89.2
K	80.3	–	–	74.0
Mg	75.3	74	94.6	75.6
Mn	83.2	68.8	95.0	89.7
Na	79.4	79.4	–	84.5
Ni	–	90.7	94.7	79.2
Zn	83	69	94.8	82.2

### 3.3 *mebipred* predicts protein metal-binding propensity from short fragments

We extracted a set of 101 054 024 50-residue peptides from the PDB protein sequences (Materials and methods); these correspond to the typical lengths of peptides that could be generated by translating DNA reads produced by next-generation sequencing (Jünemann *et al.*, 2013). We predicted metal-binding for these fragments using *mebipred* and aligned them (via BLAST) to PDB sequences following the same procedure as for complete proteins (Materials and methods; excluding hits to self). *mebipred* outperformed BLAST (Fig. 1B) in identifying peptides generated from metal-binding proteins. BLAST is not designed to deal with short-sequence alignments (Altschul *et al.*, 1990; Campagna *et al.*, 2009) and our results suggest that sequence identity may not be an accurate indicator of metal-binding either. Note that it is still possible that other alignment methods or substitution matrices, i.e. penalizing substitutions of residues often involved in metal-binding, could yield better results.

### 3.4 Ion binding preferences are consistent per Pfam family

We ran *mebipred* on the 607 903 Pfam proteins (8207 families) whose structures are available in the PDB. For 61% of the families, either all member proteins were predicted to be metal-binding or none were (Supplementary Data: stats_with_id). Of per metal predictions, 69% were cases where no members of one family bind that metal and 5% were cases where all of members of one family bind it—a total of 74% agreement of per ion predictions for members in the same family. Our results indicate that metal-binding preferences are mostly consistent within a Pfam family. This is expected, as Pfam domains reflect homology that often suggests similar functionality (Sharma *et al.*, 2019). Specifically, as we expect Pfam domains to be sequence similar, we also anticipate sequence-based models to make similar predictions for all members of a given domain. However, different ion preferences for a quarter of the families also suggest that specific metal availability within individual environments may have driven divergent evolution of new ligand-binding functionalities across organisms (Rausell *et al.*, 2010). Note that prediction error and cambialistic activity (i.e. ability to bind multiple ions) of certain proteins, which is not captured by this summary of ion binding, could also contribute to this discrepancy in metal-binding preferences of single family members.

### 3.5 *mebipred* predictions do not always reflect existing annotations of metal-binding

We compared our METAL and NO_METAL datasets with Swiss-Prot metal-binding annotations. Of the 253 377 PDB sequences mapped to Swiss-Prot [PDBSWS (Martin, 2005); April 2021], 53 652 (∼20%) had annotations that disagreed with ours. Of these 32 802 were in our METAL set, i.e. in a PDB structure with a metal ion within 5 Å of the chain but were not described as metal-binding by Swiss-Prot. Manual examination of 10 randomly chosen discrepancies confirms that the metal ion is present in a functional pocket, suggesting that Swiss-Prot annotations are incomplete. The remaining 20 850 sequences were described in Swiss-Prot as metal-binding but were not in our METAL set.

We ran *mebipred* on these 20 850 PDB–Swiss-Prot discrepancies. Our predictions (binary metal-binding at default cutoff) agreed with Swiss-Prot annotations two-thirds of the time (64%, 13 374 sequences, predicted metal-binding) even though this was in opposition of the PDB-based *mebipred’*s training data and would thus be considered a false positive. Crystal structures of metal-binding sequences may not contain a metal for a number of reasons, including biologically irrelevant binding, i.e. a metal can be bound by a protein, but is not under physiological conditions (Pidugu *et al.*, 2017), or experimental/technical crystallization decisions (Laganowsky *et al.*, 2011). However, we expect that the 1302 (6% of 20 850) non-metal-binding chains from metal ion-containing PDB structures are most likely to be true non-binders of that ion. In fact, *mebipred* predictions for these proteins agreed with PDB 41% of the time (540 sequences predicted to be non-binding)—a somewhat better agreement (versus 36%) than that for other designated metal non-binders.

A closer inspection further informs the reasons for database annotation differences. For example, 32 of the 540 predicted non-metal-binding PDB chains map to the Rieske subunit of cytochrome BC1—an Fe–S cluster binding protein (Swiss-Prot ID: Q5ZLR5) (Zhang *et al.*, 1998). None of these 32 chains, however, are complete sequences of the protein and none contain the part of the structure that would bind the Fe–S cluster. In this particular case, the annotation discrepancy arises from a technical decision not to crystallize the metal-binding regions (Zhang *et al.*, 1998). While this level of scrutiny for every disagreement between databases is beyond the scope of this work, we note that an annotation discrepancy does not necessarily constitute a ‘bug’ but, rather, a feature of the method; i.e. *mebipred* could be used to resolve database annotation conflicts.

### 3.6 *mebipred* can predict metal-binding from metagenome read translations

We compared the metal-binding profiles of the Black Sea metagenomic samples obtained at different depths in a water column (Cabello-Yeves *et al.*, 2020) (Supplementary Data: counts_sra), extracted from NCBI-SRA (Leinonen *et al.*, 2011) and processed as in Materials and methods. The relative frequencies of the resulting metal-binding protein/peptide predictions from the assembled (*p*) and unassembled (*q*) data were very similar (Supplementary Table S3); Euclidean distance (*p, q*) = 0, where *n* ∈ (Ca, Co, Cu, Fe, K, Mg, Mn, Na, Ni, Zn) indicates identical metal-binding frequency profiles (Equation 2). This result suggests that *mebipred* can reliably predict metal-binding from translations of metagenomic reads (Materials and methods).

(2) $$\text{Euclidean Distance }\left(p,q\right)=\;\sqrt{{\left(p_{1}-q_{1}\right)}^{2}+{\left(p_{2}-q_{2}\right)}^{2}+\ldots +{\left(p_{10}-q_{10}\right)}^{2}}.$$

### 3.7 Diversity of metal-binding proteins highlights environmental differences

Across a few environmental samples, we observed protein metal-binding signatures consistent with environmental features and subtypes.

#### 3.7.1 Black Sea water column

**Fig. 2.**
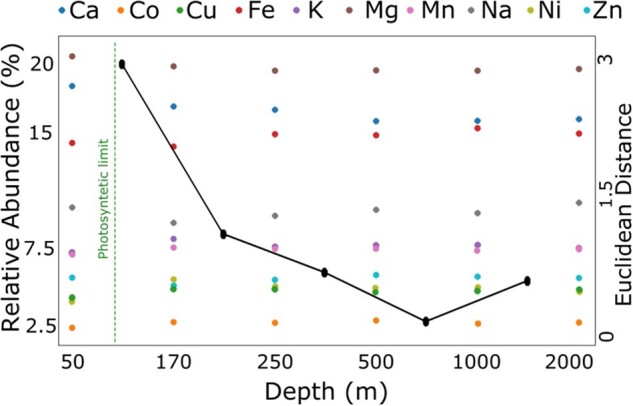
Prediction of metal-binding in Black Sea microbiomes. The points on the graph indicate the relative abundance of ion-binding proteins (left *y*-axis) predicted from metagenomic samples collected at different depths of the Black Sea (*x*-axis). The black line represents the Euclidean distance (right *y*-axis) between the vectors of predicted abundances at sequential depths; line markers are placed between the depth measurements in each comparison. Samples show a phase transition (large Euclidean distance) at the photosynthetic limit (60–100 m) (Callieri *et al.*, 2019; Gorlenko *et al.*, 2005)

From the above analysis, we observed that the percentage of reads predicted as metal-binding was ∼1% for all Black Sea samples (Supplementary Table S4). The Black Sea is a heavily stratified body of water, where pH, oxygen and light gradients have been characterized (Stanev, 1990). The sea surface layers where photosynthesis can occur, i.e. the epipelagic zone, are, by definition, up to 200 m in depth; on the Black Sea, however, almost no photosynthetic activity can be found below 100 m (Callieri *et al.*, 2019). The epipelagic zone samples in our set are slightly enriched (2% increase) in Mg-binding proteins (Fig. 2) in line with the use of Mg in chlorophyll (Chu, 1942).

In non-photosynthetic environments, we observed a trade-off between the enrichment of Mg and Fe binding proteins, which can be accounted for by the lower pH increasing Fe availability and by the abundance of iron-reducing organisms at greater depths (Canfield *et al.*, 1996). The maximal difference between the abundances of predicted metal-binding proteins is observed between the samples taken at depths of 50 and 170 m, i.e. bypassing the photosynthetic limit; as indicated by the steep slope of the line tracing the Euclidian distance between metal-binding protein abundance vectors of individual samples (Fig. 2). Sample metal-binding preferences appear more similar below 170 m (lower absolute value of slope). The difference between consecutive depths until 1000 m is in line with the changes in the environment described by the pH chemocline, changes in reduction potential, and reduced light (Jørgensen *et al.*, 1991); i.e. the deeper one goes the lower the pH, the less calcium, and the more Fe (Lewis and Landing, 1991). The change in the sign of the slope indicating increasingly different samples at 1000 and 2000 m likely accompanies a change in the microbial community (Cabello-Yeves *et al.*, 2020). This may reflect the transition from the Mesopelagic (200–1000 m), where some light and oxygen are still available, to the Antropelagic region (1000–4000 m), where there is not any of either. Alternatively, this change can highlight the fact that 2000 m is essentially the seafloor (Karatay, 2007).

#### 3.7.2 Hot spring sediments

We further analyzed 16 metagenomic samples from hot spring sediments obtained from NCBI-SRA DB (Supplementary Data: counts_sra) and described in Fullerton *et al.* (2021). The proportion of genetically encoded proteins binding each metal was similar (within 2%) for all samples (Supplementary Table S5). We observed a significant correlation between the relative frequency of proteins binding iron and the iron environmental concentrations (Pearson *r* = 0.54 *P* = 0.03; Supplementary Tables S5 and S6); for zinc and manganese, the correlation was positive, but not significant (Pearson *r* = 0.1 and 0.18, *P* = 0.71 and 0.05, respectively). Copper and nickel binding proteins, on the other hand, had a negative correlation (not significant) with the corresponding environmental concentrations (Pearson *r* = −0.1/*P* = 0.7 and Pearson *r* = −0.43/*P* = 0.1, respectively). Note that only the abundance of iron-binding proteins was significantly correlated with the environmental concentrations.

We lack complete information about metal requirements for different microbial strains. There is evidence that metabolites reflect the microbial community composition by altering the abundance of metabolite-relevant genes (Mallick *et al.*, 2019)—a finding only somewhat in line with our observations. However, why did only iron (Fe) concentrations significantly correlate with iron-binding protein abundance? Fe is considered a major element (>1000 p.p.m.), while others (Zn, Mn, Cu, Ni) are trace elements (<100 p.p.m.) (Scherer *et al.*, 1983). Metabolic requirements for each metal vary across organisms. However, iron is essential for nearly all of them; e.g. restricting iron availability to microbial invaders is part of the innate immune response (Ganz, 2009). Additionally, of the five measured metals, Fe is the only one that is present in the sampling sites at concentrations (observed: 3–400 p.p.m.) below the what is needed for growth of metal requirement annotated bacteria (Rouf, 1964; Scherer *et al.*, 1983) (average requirement: 5400 p.p.m.); in fact, bacteria aim to actively accumulate Fe using specialized proteins (Braun and Hantke, 2011). The other four metals are usually required in concentrations (Rouf, 1964) below those observed in this study. Moreover, higher concentrations may be deleterious to organism fitness, particularly for the anticorrelated metals. For example, nickel is required in trace quantities (Chivers, 2015) and competes with Mg and Ca for binding sites (Yang and Black, 1994); in high concentrations, it can also damage DNA (Sunderman Jr, 1989). Copper is frequently toxic for bacteria at environmental concentrations (Dupont *et al.*, 2011) and is thus tightly regulated. Thus, given its key role in metabolism and limiting factor status, iron concentrations could drive microbial selection and explain the abundance of genes encoding iron-binding proteins.

#### 3.7.3 Human-host microbiomes

**Fig. 3.**
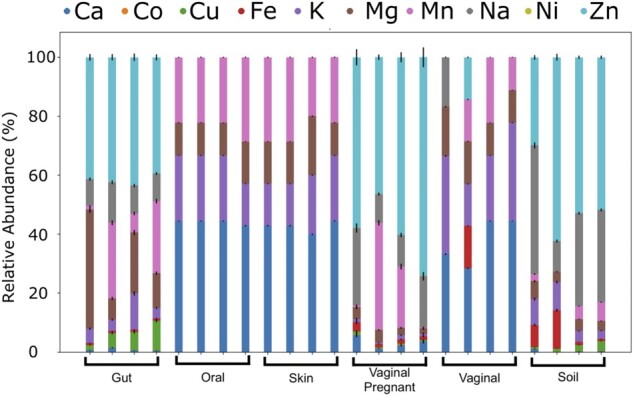
Differential abundance of metal-binding proteins across environments. Each bar represents the relative abundance of predicted metal-binding proteins (*y*-axis) in a metagenomic sample (four per environment; *x*-axis). Concentration of these proteins per environment (column colors and sizes) is similar within and is different across environments, suggesting signature metal ion preferences

We further used *mebipred* to analyze randomly chosen human host and soil microbiome samples from the NCBI-SRA DB (Supplementary Data: counts_sra). Predicted metal-binding proteins (Fig. 3) are in line with the available metals in each environment. For example, few or no iron-binding proteins are predicted in samples of human origin except for one vaginal sample, where the occurrence may be explained by menstrual cycle bleeding. Low concentrations of iron-binding proteins are observed in the gut and pregnancy-associated vaginal microbiota, both of which may be accounted for by minor bleed episodes. As mentioned above, iron sequestering is part of normal human immune response and is lethal to most pathogenic bacteria (Ganz, 2009); normal non-pathogenic microbiota are likely to be adapted to low iron environment (Yilmaz and Li, 2018). metal-binding proteins predicted to occur in the soil and in gut samples target more different metals than do skin, mouth and vaginal samples, likely due to the metabolic diversity of the former (Fierer, 2017). The predicted metal-binding proteins in skin samples target metals (Ca, K, Mg, Mn) that can be found in sweat in relatively high concentrations (>1 mg/l) (Robinson and Robinson, 1954). Other metals (e.g. Zn, Cu) are present in sweat in trace concentrations (<1 mg/l) (Cohn and Emmett, 1978; Saraymen *et al.*, 2004) and, thus, few proteins binding these metals are predicted (<1% of predictions). Furthermore, the differences in metal-binding protein abundances between vaginal samples from pregnant and non-pregnant women could reflect the large pregnancy-associated changes in the vaginal microbiome (Romero *et al.*, 2014).

*mebipred* is an advance in the field of function prediction from protein sequence, which we showed to be applicable to the annotation of metagenomic samples. It can help resolve database annotation errors and shows potential for linking function with environmental conditions. We further expect that as more metal-binding protein structures are resolved, our method can be improved and expanded, for example to the detection of other metal ions. Its capacity to annotate metal-binding informs the descriptions of microbiome diversity and environmental conditions. Finally, since most enzymes are metal-binding proteins, it could also help enzyme prospecting.

## 4 Conclusion

Here, we compiled a gold-standard experimentally derived metal-binding protein set and built *mebipred—*a sequence-based neural network predictor of metal-binding. To the best of our knowledge, *mebipred* is the only reference-free sequence-based method for identifying protein metal-binding. *mebipred* significantly outperforms existing sequence-based methods for annotation of metal-binding and can detect specific metals bound by each protein. We expect that the growth in the number of structures of metal-binding proteins will it even more powerful in the near future. *mebipred* is also faster than existing tools and can predict metal-binding using short protein fragments, making it useful in analysis of metagenomic data. In evaluation of microbiome samples, we found that differences in the number of predicted metal-binding proteins were related to the concentration of metal ions in the corresponding environments.

## Supplementary Material

btac358_Supplementary_Data
