# Supplementary material for: mebipred: identifying metal-binding potential in protein sequence
Source: Bioinformatics. 2022 May 27;38(14):3532–40. doi: 10.1093/bioinformatics/btac358 (PMC9272798; doi:10.1093/bioinformatics/btac358)
Supplement: btac358_Supplementary_Data [file btac358_supplementary_data.zip › btac358_Supplementary_Data/MEBIPRED_SOM.doc]

Supporting online material for:
*mebipred*: identifying metal binding abilities from protein sequence.

Aptekmann AA^1,2^, Buongiorno J^5^, Giovanelli D^124^, Glamoclija M^6^ , Ferreiro DU^3^, Bromberg Y^1^

SOM Table 1: number of sequence clusters binding each ion in PDB70 (redundancy reduced at 70% sequence identity).

| Ion | 5A_PDB70 |
| --- | --- |
| Ca | 1606 |
| Co | 131 |
| Cu | 167 |
| Fe | 964 |
| K | 59 |
| Mg | 1415 |
| Mn | 248 |
| Na | 790 |
| Ni | 200 |
| Zn | 1707 |
| Sum | 7287 |
| Total Metal Binding (MB)* | 5331 |

* As a protein can bind multiple ions, the total number of metal binding structures is smaller than the sum of structures binding each ion.

SOM Table 2 : *mebipred* performance on sequence clusters excluded from training.

| Maximum sequence  identity | Positive Samples | AUROC | AUPRC |  | Prec^ | Rec^ | F1_max_^ | Prec* | Rec* | F1* |
| --- | --- | --- | --- | --- | --- | --- | --- | --- | --- | --- |
| x ≤ 25% | 1970 | 0.75 | 0.74 |  | 0.58 | 0.94 | 0.72 | 0.72 | 0.63 | 0.67 |
| 25%< x ≤ 50% | 3592 | 0.72 | 0.71 |  | 0.58 | 0.92 | 0.71 | 0.65 | 0.76 | 0.70 |
| 50% < x | 29048 | 0.74 | 0.72 |  | 0.65 | 0.89 | 0.75 | 0.69 | 0.83 | 0.74 |

We aligned (via mmseq2(3) with default parameters) the 110,140 sequences excluded from training as part of “mixed clusters” to all 133,452 training sequences of the metal binding model (66,726 positives and negatives, each). We split the excluded sequences into three groups, based on the maximum sequence identity of any cluster sequence to any sequence used for training. We measured the performance using all the positives from each group and a randomly selected same size negative subsample. ^Precision, Recall, and F1 measure are computed at the cutoff resulting in maximum F1 value. *All values computed ate the 0.4 cutoff selected from cross-validation results. The difference between F1 and F1_max_ for each subset of proteins, suggests that the cutoff score can and should be varied according to the use case, e.g. the desire for better precision or better recall

SOM Table 3: Relative frequencies of predicted metal binding proteins in assembled vs unassembled metagenomic samples.

| ***Assembled*** |  |  |  |  | **%** |  |  |  |  |  |
| --- | --- | --- | --- | --- | --- | --- | --- | --- | --- | --- |
| SAMPLE (m) | Ca | Co | Cu | Fe | K | Mg | Mn | Na | Ni | Euclidean distance |
| 50 | 19.32 | 2.57 | 4.68 | 15.37 | 7.79 | 21.38 | 7.62 | 10.9 | 4.35 | 0.66 |
| 80 | 17.42 | 2.94 | 5.09 | 15.02 | 8.63 | 20.63 | 8.44 | 10.76 | 5.63 | 0.67 |
| 170 | 17.9 | 2.96 | 5.26 | 15.12 | 8.72 | 20.69 | 8.12 | 9.83 | 5.92 | 0.63 |
| 250 | 17.67 | 2.91 | 5.23 | 15.97 | 8.18 | 20.38 | 8.06 | 10.32 | 5.42 | 0.65 |
| 500 | 16.87 | 3.07 | 5.11 | 15.92 | 8.23 | 20.41 | 8.01 | 10.75 | 5.41 | 0.41 |
| 1000 | 16.91 | 2.95 | 5.11 | 16.4 | 8.28 | 20.37 | 7.96 | 10.51 | 5.4 | 0.47 |
| 2000 | 17.01 | 2.93 | 5.21 | 16.03 | 8.06 | 20.49 | 8.01 | 11.22 | 5.04 | 0.68 |
| ***Unassembled*** |  |  |  |  | **%** |  |  |  |  |  |
| SAMPLE (m) | Ca | Co | Cu | Fe | K | Mg | Mn | Na | Ni | Euclidean distance |
| 50 | 19.04 | 2.60 | 4.70 | 15.60 | 7.59 | 20.89 | 7.65 | 10.77 | 4.24 | 0.66 |
| 80 | 17.50 | 2.92 | 5.04 | 14.93 | 8.78 | 21.17 | 8.29 | 10.44 | 5.68 | 0.67 |
| 170 | 18.24 | 2.94 | 5.32 | 15.41 | 8.83 | 20.97 | 7.98 | 10.12 | 5.91 | 0.63 |
| 250 | 17.63 | 2.83 | 5.24 | 15.89 | 8.07 | 19.82 | 8.24 | 10.10 | 5.37 | 0.65 |
| 500 | 16.81 | 3.08 | 5.15 | 15.86 | 8.01 | 20.16 | 8.15 | 10.67 | 5.25 | 0.41 |
| 1000 | 17.18 | 2.99 | 5.05 | 16.69 | 8.10 | 20.41 | 8.00 | 10.38 | 5.47 | 0.47 |
| 2000 | 17.23 | 2.97 | 5.11 | 15.70 | 7.91 | 21.01 | 7.95 | 11.16 | 5.01 | 0.68 |

SOM Table 4: Metal binding predictions for Black Sea samples

| Sample | Ca | Co | Cu | Fe | K | Depth(m) | Reads | mbp/  n_reads% |
| --- | --- | --- | --- | --- | --- | --- | --- | --- |
| SRR12347146 | 1105 | 146 | 264 | 872 | 446 | 50 | 1018328 | 0.56% |
| SRR12347144 | 7652 | 1270 | 2249 | 6484 | 3763 | 170 | 4382497 | 0.98% |
| SRR12347143 | 8009 | 1327 | 2363 | 7230 | 3706 | 250 | 4392416 | 1.03% |
| SRR12347142 | 6738 | 1217 | 2035 | 6355 | 3259 | 500 | 4103257 | 0.97% |
| SRR12347141 | 10831 | 1873 | 3251 | 10453 | 5284 | 1000 | 5967681 | 1.07% |
| SRR12347140 | 9429 | 1631 | 2920 | 8955 | 4507 | 2000 | 4153399 | 1.34% |
|  |  |  |  |  |  |  |  |  |
|  |  |  |  |  |  |  |  |  |
| Sample | Mg | Mn | Na | Ni | Zn | Depth(m) | Reads | mbp/n_reads% |
| SRR12347146 | 1223 | 436 | 621 | 248 | 348 | 50 | 1018328 | 0.56% |
| SRR12347144 | 8864 | 3472 | 4227 | 2517 | 2339 | 170 | 4382497 | 0.98% |
| SRR12347143 | 9234 | 3656 | 4684 | 2497 | 2657 | 250 | 4392416 | 1.03% |
| SRR12347142 | 8078 | 3167 | 4279 | 2153 | 2478 | 500 | 4103257 | 0.97% |
| SRR12347141 | 13033 | 5099 | 6701 | 3448 | 3920 | 1000 | 5967681 | 1.07% |
| SRR12347140 | 11464 | 4481 | 6289 | 2812 | 3342 | 2000 | 4153399 | 1.34% |

SOM Table 5: Number of predicted metal binding proteins for each hot springs sample

| **SRA** | Sample | Ca | Co | Cu | Fe | K | Mg | Mn | Na | Ni | Zn |
| --- | --- | --- | --- | --- | --- | --- | --- | --- | --- | --- | --- |
| SRS6512461 | 1 | 15266 | 2406 | 4656 | 11062 | 10947 | 18544 | 8066 | 9368 | 8459 | 4479 |
| SRS6512462 | 2 | 16315 | 2653 | 4831 | 12687 | 12400 | 20825 | 9217 | 10817 | 10124 | 5555 |
| SRS6512467 | 3 | 70528 | 11442 | 21944 | 52321 | 54663 | 87904 | 39892 | 48720 | 43242 | 20696 |
| SRS6512479 | 4 | 17217 | 2810 | 4926 | 13957 | 12540 | 22162 | 9056 | 10136 | 10171 | 6471 |
| SRS6512459 | 5 | 27799 | 4642 | 8670 | 20903 | 20750 | 34372 | 15629 | 18244 | 16430 | 8401 |
| SRS6512473 | 6 | 43900 | 6967 | 13124 | 32821 | 31516 | 52025 | 23236 | 27547 | 24516 | 12728 |
| SRS6512468 | 7 | 9004 | 1522 | 2728 | 6959 | 6682 | 11635 | 4958 | 5696 | 5220 | 2795 |
| SRS6512470 | 8 | 21620 | 3207 | 6050 | 14930 | 15430 | 25819 | 11488 | 13037 | 11614 | 6245 |
| SRS6512475 | 9 | 30904 | 4654 | 9007 | 22113 | 22431 | 37631 | 16327 | 18946 | 17347 | 9431 |
| SRS6512493 | 10 | 16244 | 2338 | 4585 | 11993 | 12105 | 20108 | 8788 | 10166 | 9463 | 5221 |
| SRS6512481 | 11 | 41958 | 6551 | 12330 | 30652 | 31579 | 52692 | 23292 | 27326 | 24914 | 13189 |
| SRS6512487 | 12 | 30830 | 5653 | 9601 | 24851 | 24426 | 41000 | 18344 | 21501 | 20387 | 10008 |
| SRS6512485 | 13 | 66204 | 10806 | 20108 | 51642 | 50031 | 83913 | 36891 | 43449 | 41416 | 21723 |
| SRS6512489 | 14 | 9020 | 1557 | 2978 | 7225 | 7125 | 11678 | 5463 | 6355 | 5901 | 2976 |
| SRS6512471 | 15 | 16376 | 2693 | 4917 | 11999 | 12811 | 20867 | 9406 | 11525 | 10197 | 4978 |
| SRS6512483 | 16 | 10862 | 1933 | 3349 | 8782 | 8451 | 14443 | 6037 | 7167 | 6737 | 3498 |

SOM Table 6: Ion concentrations for each hot springs sample

| **Sample** | **Cu (ppm)** | **Cu (mM)** | **Fe (ppm)** | **Fe (mM)** | **Mn (ppm)** | **Mn (mM)** | **Ni (ppm)** | **Ni (mM)** | **Zn (ppm)** | **Zn (mM)** |
| --- | --- | --- | --- | --- | --- | --- | --- | --- | --- | --- |
| 1 | 196.57 | 3.12 | 218.3 | 3.97 | 674.09 | 11.43 | 25.14 | 0.43 | 148.44 | 2.28 |
| 2 | 30.21 | 0.48 | 360.48 | 6.55 | 2320.79 | 39.34 | 9.23 | 0.16 | 900.45 | 13.85 |
| 3 | 78.41 | 1.24 | 34.4 | 0.63 | 578.45 | 9.8 | 9.41 | 0.16 | 45.49 | 0.7 |
| 4 | 72.19 | 1.15 | 153.78 | 2.8 | 761.27 | 12.9 | 36.04 | 0.62 | 165.33 | 2.54 |
| 5 | 227.86 | 3.62 | 57.59 | 1.05 | 3434.01 | 58.2 | 36.01 | 0.62 | 100.59 | 1.55 |
| 6 | 81.8 | 1.3 | 259.85 | 4.72 | 1273.41 | 21.58 | 20.2 | 0.35 | 87.7 | 1.35 |
| 7 | 59 | 0.94 | 3.3 | 0.06 | 8.01 | 0.14 | 1.17 | 0.02 | 6.6 | 0.1 |
| 8 | 102.48 | 1.63 | 68.03 | 1.24 | 787.16 | 13.34 | 72.87 | 1.26 | 106.12 | 1.63 |
| 9 | 138.49 | 2.2 | 102.91 | 1.87 | 1865.37 | 31.62 | 110.8 | 1.91 | 145.92 | 2.24 |
| 10 | 159.93 | 2.54 | 122.19 | 2.22 | 1665.15 | 28.22 | 118.06 | 2.04 | 144.4 | 2.22 |
| 11 | 69.53 | 1.1 | 64.26 | 1.17 | 1065.7 | 18.06 | 55.82 | 0.96 | 96.57 | 1.49 |
| 12 | 10.99 | 0.17 | 403.71 | 7.34 | 3286.53 | 55.7 | 1.61 | 0.03 | 15.73 | 0.24 |
| 13 | 10.99 | 0.17 | 403.71 | 7.34 | 3286.53 | 55.7 | 1.61 | 0.03 | 15.73 | 0.24 |
| 14 | 55.87 | 0.89 | 66.22 | 1.2 | 1167.52 | 19.79 | 17.25 | 0.3 | 97.92 | 1.51 |
| 15 | 102.48 | 1.63 | 68.03 | 1.24 | 787.16 | 13.34 | 72.87 | 1.26 | 106.12 | 1.63 |
| 16 | 255.49 | 4.06 | 118.14 | 2.15 | 2243.36 | 38.02 | 34.34 | 0.59 | 532.13 | 8.19 |

**FIGURES**

**SOM Figure 1. Distribution of metal binding sequences within sequence clusters.** For most of the clusters (33,911 of 42,085) either 98% of the sequences within a cluster belong to the METAL (5,333 clusters) set or 98% of the sequences belong to the NO METAL set (28,578 clusters) .

SOM METHODS (for Figure 2 and 3) Feature extraction:

1. *Amino acid composition*: for each of the 20 standard amino acids, the percentage of the type of amino acid in the entire sequence. *Total: 20 features.*
2. *Physicochemical properties of amino acids*: We used a set of nine amino acid properties as described in Li et al. (1): hydrophobicity, hydrophilicity, number of hydrogen bonds, volume, polarity, polarizability, solvent accessible surface area, net charge index of side chains, and average amino acid mass. For each of the properties (Fig. 1), we clustered sequence residues into three categories, Low (L), Medium (M), and High (H), using “Jenks natural breaks” criterion (2); this measure seeks to minimize each class's average deviation from the class mean while maximizing each class's deviation from the means of the other classes. For each category, we further calculated its *composition* (C), *i.e.* the fraction of amino acids in the sequence belonging to the category; *transitions* (T), *i.e.* for every pair of sequential amino acids, the number of transitions from one category to another divided by sequence length; and *distribution* (D), *i.e.* the sequence position of the amino acid corresponding to category’s first occurrence, 25% of occurrences, 50%, 75%, and last occurrence, divided by sequence length. *Total: 189 features.*

**SOM Figure 2: Deriving the physicochemical features: example of “number of hydrogen bonds”.** In the toy sequence AYEAHWII, the “number of hydrogen bonds” feature can take values of 2, 3, or 4 at each amino acid. Translating each amino acid to its corresponding value and then to its category yields 23424322 and LMHLHMLL. Here we show how the property-based features *composition*, *transitions* (L-M case: Low to Medium and Medium to Low), and *distribution* (Low case) are calculated. These and the remaining physicochemical property features (*transitions* for M-H and L-H and *distributions* for Medium and High cases) are computed in the same manner.

3. For each protein structure in the PDB, individually for every metal, we identified all five amino acid-long subsequences (5mers) within 5Å of the bound metal ion (Fig. 2; i.e. for heme this means within 5Å of the Fe atom) and counted the frequency of their occurrence in all complete PDB sequences. Every query sequence is then decomposed into 5mers (via sliding window of 1) and the 5mer feature is computed as the sum of all occurrences of the individual metal-specific 5mers in the PDB dictionary. We also included as a feature the sum of the frequencies across all ions. *Total: 11 features.*

SOM Figure 3: Deriving the 5mer features. In this toy example of a protein structure that binds a metal, the red circle depicts a sphere of 5Å radius around the metal; the residues within the red circle are marked red. Each of these red residues and their neighboring residues (two on each side) make up a feature 5mer. In this example, the four 5mers are: “ISISA”, “SISAP”, “EQUEN” and “QUENC”. The query sequence (right panel) is decomposed to count the number of metal binding 5mers present. The final score for this feature is the sum of the counts of all 5mers in the sequence.

SOM Figure 4: Precision/Recall curve of a Random Forest binary classifier. An RF classifier (AUPRC = 0.42) was trained with the same dataset and using the same features as the first tier (metal binding) MBP-ANN (AUPRC=0.83). The model was generated using python sklearn RandomForestClassifier library, 100 estimators and gini impurity criterion (all default parameters: n_estimators=100, criterion='gini', max_depth=None, min_samples_split=2, min_samples_leaf=1, min_weight_fraction_leaf=0.0, max_features='auto', max_leaf_nodes=None, min_impurity_decrease=0.0, bootstrap=True, oob_score=False, n_jobs=None, random_state=None, verbose=0, warm_start=False, class_weight=None, ccp_alpha=0.0, max_samples=None).

**SOM Figure 5 Precision/Recall curve for prediction of sequences from the mixed clusters (not used in training).** We tested *mebipred* on 69,220 sequences (34,610 metal binding) in 3,883 clusters. This constitutes all of the available positives (metal binding proteins) and a randomly selected equal number of negatives from the 110,140 sequences in 8,174 clusters that were NOT used in training; i.e. these contained either: (1) less than 98% of the sequences from the METAL set, (2) less than 98% of the sequences from the NO METAL set, or (3) were from NO METAL clusters that were not used in training.

**REFERENCES**

1.Li Z, Tang J, Guo F. Identification of 14-3-3 proteins phosphopeptide-binding specificity using an affinity-based computational approach. PloS one. 2016;11(2).

2.Jenks GF. The data model concept in statistical mapping. International yearbook of cartography. 1967;7:186-90.

3.Steinegger M, Söding J. MMseqs2 enables sensitive protein sequence searching for the analysis of massive data sets. Nature biotechnology. 2017;35(11):1026-8.
